# Recent advances of transcriptomics and proteomics in triple‐negative breast cancer prognosis assessment

**DOI:** 10.1111/jcmm.17124

**Published:** 2022-02-11

**Authors:** Yuan Li, Xiangyi Kong, Zhongzhao Wang, Lixue Xuan

**Affiliations:** ^1^ Department of Breast Surgical Oncology National Cancer Center/National Clinical Research Center for Cancer/Cancer Hospital Chinese Academy of Medical Sciences and Peking Union Medical College Beijing China

**Keywords:** breast cancer, prognosis, proteomics, review, transcriptomics, triple‐negative breast cancer

## Abstract

Triple‐negative breast cancer (TNBC), a heterogeneous tumour that lacks the expression of oestrogen receptor (ER), progesterone receptor (PR) and human epidermal growth factor receptor 2 (HER2), is often characterized by aggressiveness and tends to recur or metastasize. TNBC lacks therapeutic targets compared with other subtypes and is not sensitive to endocrine therapy or targeted therapy except chemotherapy. Therefore, identifying the prognostic characteristics and valid therapeutic targets of TNBC could facilitate early personalized treatment. Due to the rapid development of various technologies, researchers are increasingly focusing on integrating ‘big data’ and biological systems, which is referred to as ‘omics’, as a means of resolving it. Transcriptomics and proteomics analyses play an essential role in exploring prospective biomarkers and potential therapeutic targets for triple‐negative breast cancers, which provides a powerful engine for TNBC’s therapeutic discovery when combined with complementary information. Here, we review the recent progress of TNBC research in transcriptomics and proteomics to identify possible therapeutic goals and improve the survival of patients with triple‐negative breast cancer. Also, researchers may benefit from this article to catalyse further analysis and investigation to decipher the global picture of TNBC cancer.

## 1 INTRODUCTION

Breast cancer is the most commonly diagnosed cancer and the leading cause of cancer death in women worldwide.^1^ Different breast cancer subtypes have distinct biological, morphological, histological, and molecular features and display different therapy responses. Triple‐negative breast cancer (TNBC), a heterogeneous subtype characterized by the absence of oestrogen receptor (ER), progesterone receptor (PR) and human epidermal growth factor receptor 2 (HER2), represents 12% to 17% of all breast carcinomas.^2^ It is often seen in younger and premenopausal women and more frequently in African‐American women.^3^ A study has shown that they preferentially metastasize to the brain and have a higher recurrence potential and often worse prognosis than other breast cancer subtypes.^4^ Given the lack of specific molecular targets, TNBC treatment is mainly based on surgery and assisted with radiotherapy and chemotherapy.^5^ Chemotherapy includes neoadjuvant chemotherapy and postoperative chemotherapy. The effect of long‐term chemotherapy is significantly reduced due to therapy resistance, which easily leads to tumour recurrence and distant metastasis.^6^, ^7^ Less than 30% of women with metastatic breast cancer will survive five years after initial diagnosis despite systematic chemotherapy and virtually all metastatic TNBC patients eventually die of this disease.^8^ Therefore, by analysing the essential characteristics of TNBC and using effective indicators to determine the clinical prognosis, it is possible to find a suitable alternative therapy by exploring the specific treatment targets.

Transcriptomic investigations have been used to investigate promised biomarkers and potential therapeutic targets for human tumours.^9^ Microarray analysis helps to measure the gene expression levels via complementary probe hybridization, and a variety of breast cancer‐related genes have been found.^10^, ^11^ Moreover, the broad utilization of RNA sequencing (RNA‐seq) technologies has dramatically expanded our knowledge of breast cancer.^12^ Utilizing RNA‐seq, we can quantify genes that are expressed at extremely low levels.^13^

Proteomics approaches have emerged as a powerful technique for performing protein profiling and discovering novel biomarkers associated with cancer.^14^ Targeted proteomics offers new strategies for validating these candidate biomarkers’ diagnostic, prognostic, or predictive performance, which can be precisely quantified in a large cohort of clinical samples. There are two types of targeted proteomics approaches: non–MS‐based methods that use protein detection antibodies (Western blot, ELISA, immunohistochemistry and reverse‐phase protein array) and MS‐based methods (mass spectrometry imaging, targeted proteomics and next‐generation proteomics). The rapid advancement of proteomics offers a unique opportunity to investigate the proteome of triple‐negative breast cancer further.

Traditionally, four different subtypes of breast cancer have been identified based on the expression of three molecules: the oestrogen receptor (ER), the progesterone receptor (PR) and the human epidermal growth factor receptor 2 (HER2). Triple‐negative breast cancer (TNBC), one of these subtypes, is characterized by the absence of all three receptors. Subtypes of breast cancer can be classified according to their molecular characteristics as (1) Basal‐like (Triple‐negative), (2) Luminal A, (3) Luminal B and (4) HER2‐positive, and additionally Normal‐like and Claudin‐low.^3^ Most basal‐like tumours are triple‐negative breast cancers, but not all.

Our current understanding of breast cancer biology is more comprehensive than ever. Various molecular subtypes of breast cancer have been elucidated using genomic profiling strategies and other major technological discoveries, opening new windows into breast cancer treatment and research. However, the basal‐like (Triple‐negative) subtype, the most distinct among all the intrinsic subtypes of breast cancer, has not been well characterized.^15^ A genomic study has also revealed that the basal‐like (Triple‐negative) breast cancer subtype is not only distinct among other breast cancer subtypes but also other cancer types.^16^ A further subclassification of this aggressive cancer subtype is urgently required to help develop more targeted treatments for TNBC patients with better clinical outcomes.

Proteomics and transcriptomics are already impacting TNBC research, and this article reviews the latest prognostic studies of TNBC through proteomics and transcriptomics to better understand TNBC and its potential therapies.

## 2 TRANSCRIPTOME ANALYSIS OF TNBC

### 2.1 Sequencing analysis of whole transcriptome

With the deep understanding of genes and the application of high‐throughput technology continues to mature, the prognosis can be determined by constructing a gene expression scoring system or typing according to the characteristics of TNBC gene expression profile.^17^ At the same time, prognostic indicators can be obtained by sequencing and analysing the whole transcriptome of each subtype of the tumour. Lehmann et al. classify TNBC into seven subtypes based on a comprehensive transcriptomic analysis of 21 data sets for breast cancer.^17^ These include two basal‐like subtypes (BL1 and BL2), an immunomodulatory subtype (IM), a mesenchymal subtype (M), a mesenchymal stem‐like subtype (MSL), a luminal androgen receptor subtype (LAR) and an unstable unclassified set (UNS). Specific genes linked to cell proliferation and DNA damage response are strongly expressed in the BL1 subtype and this subtype preferentially responds to cisplatin and poly (ADP‐ribose) polymerase (PARP) inhibitors. The BL2 subtype is enriched with genes associated with growth factor pathways, suggesting that growth factor inhibitors may be effective for the BL2 subtype. The IM subtype has abundant genes involved in immune‐mediated reactions, and programmed cell death 1/programmed death‐ligand 1 (PD1/PDL1) inhibitors are expected to be a hopeful therapeutic option for this subtype. Both subtypes of M and MSL explicitly express genes relevant to cell motility, cellular differentiation, and growth factor pathways, while the MSL subtype expresses lower proliferation genes than those present in the M subtype. For these two subtypes, the mammalian target of rapamycin (mTOR) inhibitors and targeted epithelial‐to‐mesenchymal transition (EMT) agents are candidate drugs. The LAR subtype is named for the AR enrichment, and anti‐androgen treatments (eg bicalutamide, an AR antagonist) are undergoing clinical trials.^18^ Liu et al. analysed the transcriptome data and found four molecular types, which were named FUSCC typing, including immunomodulatory (IM), luminal androgen receptor (LAR), mesenchymal‐like (MES), Basal‐like and immunosuppressive (BLIS),^19^ the first two types are basically consistent with IM and LAR in Lehmann's study.^17^

Further analysis of this study found that MES with FUSCC typing contained mesenchymal stem cell‐like (MSL) and mesenchymal (M) in Lehmann typing, while BLIS mainly included basal cell‐like 1 (BL1) and M. Survival analysis suggested that the relapse‐free survival (RFS) of BLIS type was worse than IM, LAR and MES.

Besides, the analysis of overall survival (OS) and RFS showed that the changes of differentially expressed genes were firmly related to the prognosis of the tumour. Yang et al. compared the expression profiles of mRNAs, lncRNAs and miRNAs between 111 TNBC tissues and 104 non‐cancerous tissues utilizing RNA‐Seq Data from The Cancer Genome Atlas (TCGA). Data indicated that LHX1, WISP1 and S1PR1 were inversely related to patients’ overall survival, while SORBS1 was positively correlated with OS.^20^ Liu et al. used the biomarker combination of ALDH and CD24/CD44 to sort four populations isolated from triple‐negative breast cancer (TNBC) patient‐derived xenografts, and performed whole‐transcriptome sequencing on each population. They found that in ALDH+CD24−CD44+ breast cancer stem cells, those with high expression of P4HA2 and PTGR1 and low expression of RAB40B had shorter RFS, indicating these genes might be important prognostic markers in TNBC.^21^ Chen et al. validated HORMAD1 mRNA levels were significantly upregulated in both breast cancer cell lines and clinical samples using qRT‐PCR, and survival analysis suggested that its high expression was associated with worse RFS.^22^ The conclusion was also confirmed by immunohistochemical detection and clinicopathological information analysis. Li et al. analysed the CCR7 gene amplification and mRNA expression levels and found that the prognosis of patients with positive CCR7 expression was significantly better than those with negative expression in TNBC patients.^23^ Although these studies lack uniform standards, they can be used as a clinical prognostication of tumour therapy to some extent.

### 2.2 Non‐coding RNA sequencing analysis

Most human genes have no function of coding proteins, and their transcriptional products are non‐coding RNA (ncRNA), including long non‐coding RNA (lncRNA), microRNA (miRNA), circular RNA (circRNA) and many more, which participate in the process of cell biology at different levels such as post‐transcription, translation and epigenetics.^24^

**FIGURE 1:**
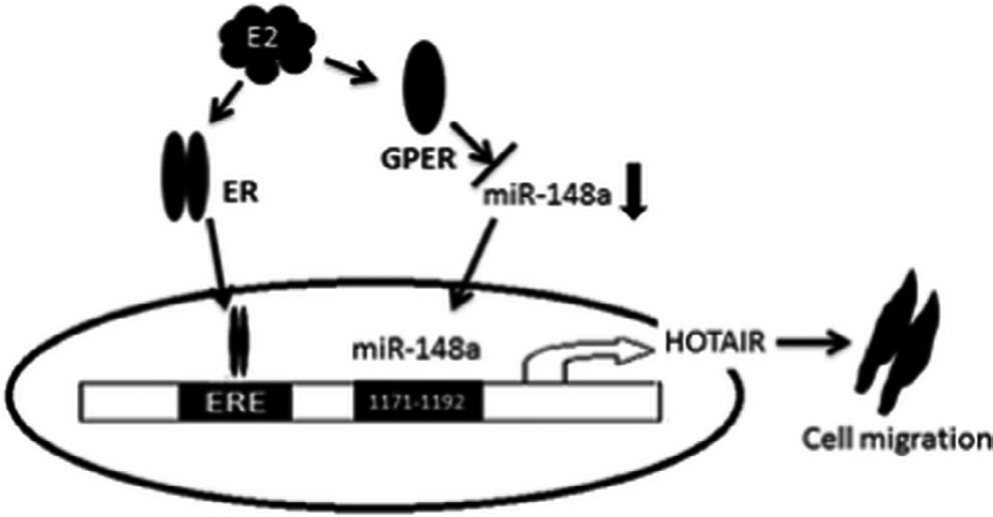
Model of E2‐induced breast cancer cell migration via upregulation of HOTAIR expression. Reprinted from.^29^ Copyright © 2015 J Transl Med

Long non‐coding RNAs (lncRNAs) are independently transcribed RNA species greater than 200 nucleotides that lack open reading frames (ORFs). Functionally characterized lncRNAs have been shown to act as transcriptional enhancers, transcription factor decoys, transcriptional guides, scaffolds for molecular interactions and competitive endogenous RNAs (ceRNAs) that sponge miRNAs and proteins and other molecules.^25^ Many lncRNAs promote cancer development, metastasis, drug resistance and abnormally expressed in various tumours and play an indispensable role in TNBC, supporting their potential clinical relevance.^26^, ^27^ Lin et al. found that both LINK‐A expression and LINK‐A‐dependent signalling pathway activation are associate with triple‐negative breast cancer, promoting breast cancer glycolysis reprogramming and tumorigenesis, leading to poor prognosis.^28^ Tao et al. showed that E2 significantly upregulated HOTAIR’s expression in MDA‐MB‐231 and BT549 triple‐negative breast cancer cells by inhibiting miR‐148a and promoting the migration of TNBC (Figure 1).^29^

**FIGURE 2:**
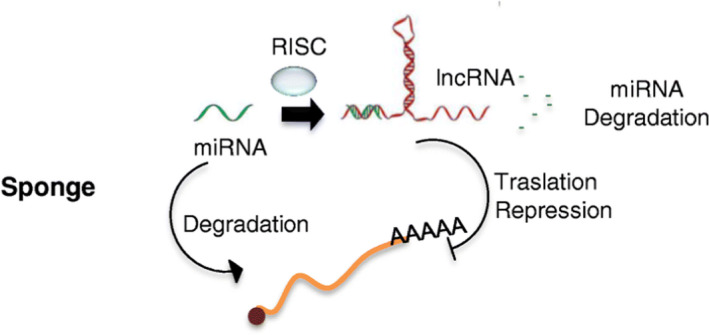
lncRNAs that by complementarity of bases succeed in matching or sequestering sequences of small non‐coding RNAs, such as miRNAs, are controlling bioavailability of miRNAs, vs. lncRNAs themselves, with the functional biological repercussions at cellular or physiological level. RNA‐induced silencing complex RISC. Reprinted from.^34^ Copyright © 2017 Biomed. Pharmacother

It is worth noting that ERRLR01 and MALAT1 are also regulated by the E2 hormone signalling pathway and participate in TNBC migration and invasion.^30^, ^31^ One study identified potential core lncRNAs in TNBC by co‐expression networks and found that the patients with low expression of potential core lncRNA‐RMST (rhabdomyosarcoma 2) had worse overall survival.^32^ Wang et al. analysed five lncRNAs (ENSG00000250337, ENSG 00000224137, ENSG00000266088, ENSG00000238121 and ENSG00000260257) models to evaluate the prognosis of breast cancer and found that the model is independent of other scoring systems, suggesting that these indicators could distinguish breast cancer subtypes.^33^ LncRNA also has a ‘sponge function’ (Figure 2), Xu et al. demonstrated that long non‐coding RNA ANRIL overexpression modulated TNBC tumorigenesis through acting as molecular ‘sponge’ for miR‐199a, participating in a variety of pathophysiological processes.^34^ Other studies also showed that LncRNA AWPPH and lncRNA POU3F3 might promote cancer cells’ proliferation in triple‐negative breast cancer, while LncRNA NEF overexpression inhibited the migration and invasion of TNBC cells.^35^, ^36^, ^37^

MicroRNA (miRNA), a class of non‐coding small RNA that post‐transcriptionally regulates gene expression, plays a vital role in cell proliferation, differentiation and apoptosis by targeting multiple downstream genes.^38^ The previous study with relatively small numbers of patients has specifically evaluated tumour miRNA markers in TNBC and showed that miRNAs play a crucial role in cellular growth and proliferation, cellular movement and migration and Extra Cellular Matrix degradation.^39^ Table 1 lists the recent studies of MicroRNAs in triple‐negative breast cancer.

**TABLE 1:** MicroRNAs reported in triple‐negative breast cancer

RNA	Target/ Axis	Endogenous expression in TNBC	Main biological function(s) in TNBC	References
LINK‐A	HIF1α	Upregulated	Glycolytic reprogramming and tumorigenesis	28
HOTAIR	E2/GPER‐miRNA148‐HOTAIR	Upregulated	Cell migration	29
ERRLR01	17β‐estradiol signalling pathway	Upregulated	Epithelial development and cellular differentiation	30
MALAT1	MALAT1‐miRNA‐1/slug	Upregulated	Cell proliferation, invasion	31
RMST	‐	Downregulated	Enhance cell apoptosis and regulate cell cycle	32
LncRNA ANRIL	MiR‐199a	Upregulated	Functions as tumour‐promoting molecular in TNBC tumorigenesis	34
LncRNA AWPPH	FZD7	Upregulated	Cell proliferation	35
LncRNA POU3F3	Caspase 9	Upregulated	Promote proliferation and inhibit apoptosis of cancer cells in triple‐negative breast cancer	36
LncRNA NEF	MiRNA‐155	Downregulated	Inhibit the migration and invasion of breast cancer cells	37
MiR‐146a / b‐5p	BRCA1	Upregulated	Cell proliferation	42
MiR‐155	VHL	Upregulated	Promote breast cancer growth and angiogenesis	44
MiRNA‐21	PTEN	Upregulated	Cell proliferation	45
MiR‐455‐3p	EI24	Upregulated	Improve cell proliferation, invasion and migration abilities in TNBC	47
MiR‐128	INSR and IRS1	Downregulated	Inhibit glucose consumption and mitochondrial energy production in TNBC cells	48
MiR‐155	RAD51	Upregulated	Promote tumour cell proliferation, angiogenesis and EMT but decreases tumour cell apoptosis	49
MiR‐212‐5p	Prrx2	Downregulated	Inhibit cell proliferation and invasion (inhibits TNBC growth and metastasis)	50
MiR‐124	ZEB2	Downregulated	Inhibit the proliferation, metastasis and epithelial‐mesenchymal transition (EMT) of TNBC cells	52
MiR‐17‐5p	ETV1	Downregulated	Inhibit TNBC cells proliferation, migration and invasion	53
MiR‐9	PDGFRβ	Upregulated	Cell migration, invasion, EMT	54
MiR‐34a	C‐SRC	Downregulated	Inhibit proliferation and invasion	55
MiR‐497	SMAD7	Downregulated	Suppress breast cancer cell proliferation and invasion in vitro	56
MiR‐1296	CCND1	Downregulated	Suppress cell proliferation and induces apoptosis in TNBC,sensitizes cells to cisplatin treatment	57
MiR‐223	HAX‐1	Downregulated	Promote TNBC cell apoptosis (enhances the anti‐tumour effect of doxorubicin and cisplatin)	58
MiR‐211‐5p	SETBP1	Downregulated	Inhibit cell proliferation and induces apoptosis,suppress breast cancer cells invasion and migration	59
MiR‐217	KLF5	Downregulated	Inhibit TNBC cell growth, migration, and invasion	60
MiRNA‐301a	PTEN‐Wnt/β‐catenin Signalling pathway	Upregulated	Cell proliferation, colony formation, migration, invasion	61
MiRNA‐454	MiRNA‐454–3'UTR‐Smad4/PTEN Signalling pathway	Upregulated	Transcription inhibition	62
MiRNA‐221‐3p	MiRNA‐221‐3p‐3'UTR‐PARP1 Signalling pathway	Downregulated	Cell migration and epithelial‐mesenchymal transformation	63
MiRNA‐34c	MiRNA‐34c‐GIT1/FRA‐1	Downregulated	Cell migration and invasion of cell cycle arrest	64
CircGFRA1	MiR‐34a	Upregulated	Promote proliferation and inhibit apoptosis in TNBC	67
CircAGFG1	MiR‐195‐5p (circAGFG1/miR‐195‐5p/CCNE1 axis)	Upregulated	Promote TNBC cell proliferation, increases TNBC cell migration and invasion and modulates cell cycle and apoptosis, functions as a sponge for miR‐195‐5p	68
Circ‐UBAP2	MiR‐661/MTA1	Upregulated	Promote proliferation and migration in TNBC	69
CircKIF4A	MiR‐375(circKIF4A‐miR‐375‐KIF4A axis)	Upregulated	Promote cell proliferation and metastasis in TNBC,acts as a sponge for miR‐375	70
CircANKS1B	MiR‐148a / 152‐3p‐USF1,TGF‐β1/ Smad	Upregulated	Promote breast cancer invasion and metastasis, serves as a sponge for miR‐148a‐3p and miR‐152‐3p	71

Considering that the overall miRNA score can better define the tumour prognosis than the single miRNA index, some studies have tried to establish a feature score system to evaluate the tumour prognosis. They systematically evaluated 57 metastasis‐related miRNAs in tumour tissue in 456 TNBC patients and proved that the expression levels of miR‐374b‐5p, miR‐27b‐3p, miR‐126‐3p and miR‐218‐5p in tumour tissues predict TNBC outcomes.^40^ Avery‐Kiejda et al. have identified 27 miRNAs related to the metastatic capabilities of TNBC cells.^41^ The expression of some miRNAs in TNBC is upregulated and may serve to promote the growth and/or invasion of TNBC cells. Therefore, this type of miRNAs is referred to as oncomiRs, including miR‐146a/146b,^42^ miR‐181a/181b,^43^ miR‐155,^44^ miR‐21,^45^ miR‐720^46^ and miR‐455.^47^ Xiao et al. examined the expression of miR‐128 in 110 TNBC patients and demonstrated that miR‐128 was able to inhibit the proliferation of TNBC cells.^48^ Jang et al. proved that miR‐9 overexpression was significantly associated with poor disease‐free survival and distant metastasis‐free survival (DMFS) in TNBC, while the high level of miR‐155 expression showed significant association with better DMFS.^49^ Lv et al. investigated the functional role of miR‐212‐5p in TNBC and found that miR‐212‐5p inhibits cell migration and invasion of TNBC during cancer progression.^50^ Other microRNAs such as miR‐493, miR‐124 and miR‐17‐5p are protentional prognostic factors in triple‐negative breast cancer. High expression of miR‐493, miR‐124 and miR‐17‐5p were all associated with better outcomes.^51^, ^52^, ^53^

Conversely, some other miRNAs are decreased in TNBC and can function as tumour suppressors to inhibit cancer cell growth, induce apoptosis and reduce metastasis. These miRNAs are defined as anti‐oncomiRs, including the miR‐200 family,^54^ miR‐34a,^55^ miR‐497,^56^ miR‐1296,^57^ miR‐223,^58^ miR‐211^59^ and miR‐217.^60^ The high expression of miRNA‐301a and miRNA‐454 and the low expression of tumour suppressor miRNAs such as miRNA‐221‐3p and miRNA‐34c all indicate poor prognosis of TNBC.^61^, ^62^, ^63^, ^64^ Studies of the systemic delivery of miRNA mimics or inhibitors via nanotechnologies are ongoing and hold great promise for cancer management.^65^

**FIGURE 3:**
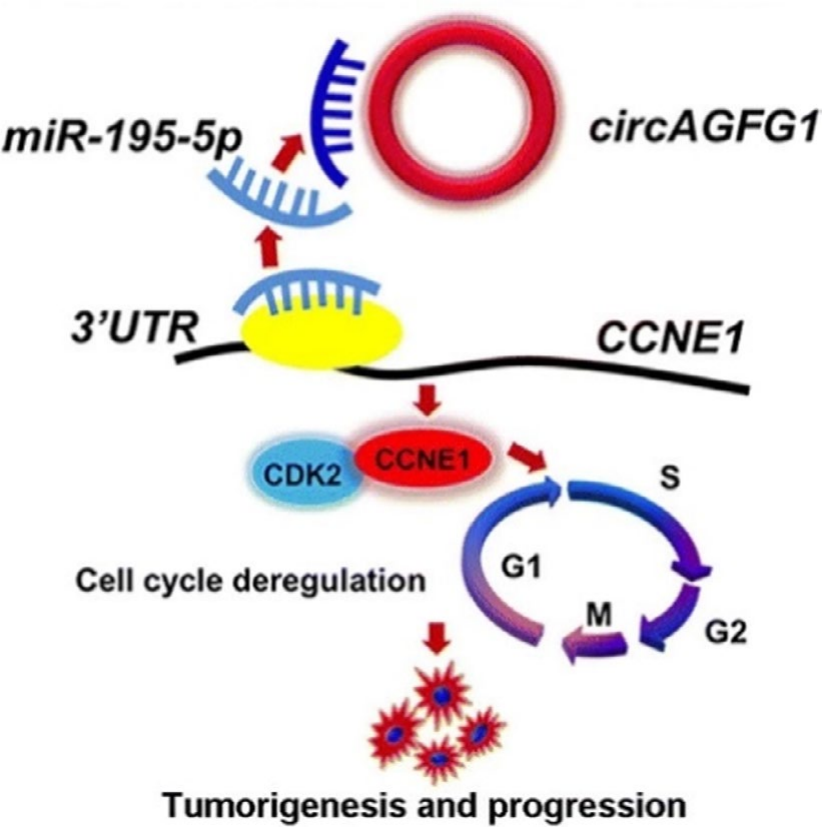
Schematic diagram of how circAGFG1 promotes TNBC tumorigenesis and progression. Reprinted from.^68^ Copyright © 2019 Mol. Cancer

Circular RNAs (circRNAs), a new type of endogenous non‐coding RNA, have the characteristics of a continuous covalently closed loop without the 5′‐cap structure and the 3′‐poly‐A tail.^66^ Some studies have revealed that circRNAs are associated with TNBC and can be the potential prognosis marker for TNBC. He et al. showed that circGFRA1 functions as a competing endogenous RNA (ceRNA) to regulate GFRA1 expression through sponging miR‐34a to promote proliferation and inhibit apoptosis in TNBC, which correlated with reduced survival of patients.^67^ Yang et al. demonstrated that circAGFG1 could sponge miR195‐5p to modulate CCNE1 expression, leading to tumorigenesis and development of TNBC (Figure 3).^68^ Wang et al. revealed that circ‐UBAP2 (hsa_circ_0001846) was markedly upregulated in TNBC, and its expression was associated with unfavourable prognosis. They noticed that circ‐UBAP2 was able to sponge miRNA‐661 to increase the expression of the oncogene MTA1, serving as a promising therapeutic target for TNBC patients.^69^ Tang et al. explored the regulatory mechanisms of circKIF4A in TNBC and found that circKIF4A was dramatically upregulated and positively associated with poorer survival of TNBC. CircKIF4A regulated the expression of KIF4A via sponging miR‐375 and acted as a prognostic biomarker for TNBC.^70^ These studies all indicated that circRNAs could act as the ‘sponge’ of miRNA, forming the corresponding circRNA‐miRNA‐mRNA axis and exerting the function of endogenous competitive RNA (ceRNA), such as interacting with RNA‐binding protein, regulating transcription factors, alternative splicing and translation, and participate in tumour proliferation, invasion and metastasis.^67^, ^68^, ^69^, ^71^

Zeng et al. found that the upregulated circANKS1B expressed in TNBC can increase the expression of transcription factor USF1, upregulated TGF‐1 and activated TGF‐β1 / Smad signal transduction through Mirna‐148a‐3p and MIRNA‐152‐3P to induce EMT to promote breast cancer invasion and metastasis.^71^ Its expression is closely related to lymph node metastasis and advanced clinical stage and function as an independent risk factor for OS in breast cancer patients. The study further confirmed that ESRP, which promotes circANKS1B synthesis, is regulated by USF1, suggesting that circRNA and mRNA are not linearly regulated.

### 2.3 Single‐cell RNA sequencing analysis

Sunny Wu et al. used a single‐cell RNA sequencing strategy to sequence nearly 24,300 single cells from five patients with triple‐negative breast cancer.^72^ By differing gene expression patterns, they identified two cancer‐associated fibroblasts (CAF) as well as two subpopulations of perivascular‐like (PVL) cells. After delving into these stromal clusters, they began to sort out certain microenvironmental changes that might affect tumour growth or treatment response. In particular, the researchers noted that an inflammatory cancer‐associated fibroblast (iCAF) releases the chemokine CXCL12, a signalling molecule that inhibits the anti‐tumour activity of T cells. They believe that this promises to point the way to enhanced immunotherapy for triple‐negative breast cancer.

Mihriban et al. also sequenced >1500 cells from six female patients with primary triple‐negative breast cancer using single‐cell RNA sequencing in order to investigate the underlying biology of triple‐negative breast cancer.^73^ Through computational analysis of individual tumour cells and the subpopulations they contained, they found that the heterogeneity of gene expression programs among each tumour cell was variable and largely correlated with the clonality of inferred genomic copy number changes, suggesting that genotype drives the gene expression phenotype of individual subpopulations. This analysis reveals functional heterogeneity in TNBC and its association with genomic evolution, and uncovers unanticipated biological principles that contribute to the poor prognosis of the disease.

Long non‐coding RNAs have been shown to play an important role in TNBC as increasingly important oncological targets. Pinkney et al. used single‐cell RNA sequencing to study the expression of lncRNAs in TNBC.^74^ They found that single‐cell RNA sequencing could identify the level of expression of lncRNAs in an aggressive subpopulation of xenograft tumours, and their finding that lncRNAs are expressed at low levels in TNBC xenograft cells may help us identify new therapeutic targets in future.

Triple‐negative breast cancer is a type of malignant breast cancer that is often resistant to chemotherapy. However, the cause of this resistance is due to selection of pre‐existing clones by chemotherapy or to genetic mutations occurring during chemotherapy has not been determined. To address this question, Charissa et al. used total exome sequencing and single‐cell DNA and RNA sequencing to study genetic and expression changes in 20 TNBC patients during neoadjuvant chemotherapy.^75^ They found that neoadjuvant chemotherapy resulted in the disappearance of cancer clones in 10 patients, while clones in another 10 patients persisted during treatment. They performed a more in‐depth and detailed analysis of eight patients using single‐cell DNA sequencing and single‐cell RNA sequencing analysis, which showed that drug‐resistant cancer cells were present prior to treatment and were adaptively selected for by neoadjuvant chemotherapy, while the gene transcriptional profiles of cancer cells in these patients underwent reprogramming after chemotherapy. With follow‐up studies, we may be able to predict which patients are likely to benefit from chemotherapy, thereby improving the precision of treatment.

## 3 PROTEOMIC ANALYSIS OF TNBC

The difference between RNA and protein expression levels prevents functional biological characteristics from fully reflecting gene expression characteristics. Therefore, functional proteomics analysis is used as supplementary information, and the integration of genomic and transcriptome data is conducive to the discovery of new targets.^76^ Intrinsically, proteins are more complex, dynamic, and reflect biological function more closely than genes. The requirements for protein analysis were also exemplified by shortcomings in the bioinformatics capacity to predict gene products’ presence and function. The requirement for protein analysis was also exemplified by shortfalls in bioinformatics’ capacity to predict the presence and function of gene products. A single gene can encode several distinct proteins, as a single pre‐mRNA transcript can be spliced into various isoforms of proteins and modified in various ways (modifications known as post‐translation modifications or PTMs) after translation. The emerging technology of modern proteomics allowed us to study protein abundance, protein‐protein interactions, PTM and ultimately protein function. Cuzick et al. proposed the IHC4 scoring standard, which confirmed its prognostic significance and showed that proteomics research on TNBC is one of the directions worth exploring.^77^ Relevant studies investigating triple‐negative breast cancer‐associated proteins using proteomics methods are listed in Table 2.

**TABLE 2:** Proteins associated with TNBC by using proteomics

Protein	Samples	Mass spectrometry	Quantitation method	Status	Potential clinical use	References
Gαh (Gαh‐PLCδ1 signalling axis)	Cell lines	Immunoblotting	Non‐MS	Upregulate	Prognostic biomarker	78
EpCAM	Tissue	IHC	Non‐MS	Upregulate	Prognostic biomarker	79
CPA4	Tissue	IHC	Non‐MS	Upregulate	Prognostic biomarker	80
TIMP‐1	Tissue	Western blot and ELISA assays	Non‐MS	Upregulated	Prognostic biomarker	81
PAI‐1	Tissue	IHC	Non‐MS	Upregulated	Prognostic biomarker	82
SPAG5	Tissue	IHC	Non‐MS	Upregulated	Prognostic biomarker	83
GGNBP2	Tissue	IHC	Non‐MS	Downregulated	Prognostic biomarker	84
CYPOR	Tissue	IHC	Non‐MS	‐	Prognostic biomarker	85
NF‐κB	Cell lines	iBAQ(absolute quantitation)	LC‐MS / MS	Upregulated	Prognostic biomarker	92
DP,TPS1,TrpRS	Tissue	iTRAQ labelling	MALDI‐MS/MS	Upregulated	Prognostic biomarker, drug target	93
A2M	Tissue and serum	iTRAQ labelling	‐	Upregulated	Prognostic biomarker	94
C4BPA	Tissue and serum	iTRAQ labelling	‐	Downregulated	Prognostic biomarker	94
MCM5, STMN1, GLS, RCL1, C9ORF114, ENO1	Cell lines	Super‐SILAC labelling	LC‐MS/MS	Upregulated	Prognostic biomarker	95
AGR2, MLPH, HID1, CMBL, FOXA1	Cell lines	Super‐SILAC labelling	LC‐MS/MS	Downregulated	Prognostic biomarker	95
ENO1	Cell lines	Label‐free quantification	LC‐MS/MS	Upregulated	Prognostic biomarker	100
TRIM28, HSP90‐alpha, hnRNP A1, CLTC, and myosin‐9, HDGF,	Tissue	Label free	LC‐LTQ/FT‐ICR MS	Phosphorylated	Drug target	101
HSP90, AB1, PTRF1, AHNAK, SEPT2	Tissue	Label free	LC‐LTQ/FT‐ICR MS	Dephosphorylated	Drug target	101
CMPK1, AIFM1, FTH1, EML4, GANAB, CTNNA1, AP1G1, STX12, AP1M1, CAPZB, MTHFD1	Tissue	Label free	Nanoscale liquid chromatography and tandem mass spectrometry (nLC‐MS/MS)	Upregulated	Prognostic biomarker	96
MTHFD1	Tissue	Label free	nLC‐MS/MS	Downregulated	Prognostic biomarker	96
FTH1	Tissue	Label free	nLC‐MS/MS	Upregulated	Prognostic biomarker	97
PTPN12	Cell lines	Dimethyl labelling	LC‐MS/MS	Downregulated	Prognostic biomarker	98
HSP70 kDa‐8, periostin, RhoA, actinin alpha 4, cathepsin D, preproprotein, annexin 1, ALDH1A1, G3BP	Tissue	Label free	LC‐MS/MS	Upregulated	Prognostic biomarker, drug target	99
Thymosinβ4, Transketolase, Transferrin	Tissue	Label free	LC‐MS/MS	Downregulated	Prognostic biomarker, drug target	99
AXL	Cell lines	SILAC	MS	Upregulated	Prognostic biomarker	102

### 3.1 Non‐mass spectrometry‐based proteomic platforms in TNBC

Non‐MS techniques such as Western blotting, enzyme‐linked immunosorbent assay (ELISA), immunohistochemistry (IHC), immunocytochemistry (ICC), and recently protein microarray (PMA) and tissue microarray (TMA) analyses, all require specific antibodies to examine target proteins. Many of these non–MS‐based technologies are beneficial for addressing particular problems.

Huang et al. estimated the prognostic values of Gαh and PLCδ1 by performing immunohistochemical staining experiments and showed that Gαh is a poor prognostic marker and correlates with the metastatic evolution of TNBC cells. Moreover, clinicopathological analyses revealed that the combined signature of high Gαh/PLCδ1 levels leads to worse prognosis in TNBC patients. This study established Gαh/PLCδ1 as a poor prognostic factor for TNBC patients.^78^ Agboola et al. detected the expression of EpCAM in TNBC by immunocytochemistry and found that it was positively correlated with tumour size and grade, and the disease‐free interval (DFI) and metastasis‐free survival (MFS) of this type of patients were significantly shortened.^79^ Handa et al. detected CPA4 expression in TNBC patients by immunocytochemistry, suggesting that the OS and DFS of patients with high CPA4 expression were significantly shorter, and the results of multivariate analysis showed that CPA4 was an independent prognostic factor for poor survival.^80^ Cheng et al. analysed the expression level of TIMP‐1 using Quantikine Human TIMP‐1 ELISA Kits in breast cancer tissues and found that serum TIMP‐1 levels were strongly enhanced in TNBC patients, which lead to a poor prognosis of TNBC patients.^81^ Other studies also used non‐MS techniques showed that the high expression of PAI‐1, SPAG5 and CYPOR were associated with the prognosis of patients with TNBC, whereas GGNBP2 suppressed cell proliferation, migration and invasion.^82^, ^83^, ^84^, ^85^

Despite significant impacts on cancer research, the widespread application of non‐MS techniques can be limited by the availability or high cost of producing suitable antibodies.

MS is one of the most versatile and useful tools in cancer research among proteomic tools. Proteomic research based on MS has enabled worldwide analyses of proteomes that have led to the discovery of new protein signatures for breast cancer.

### 3.2 Quantitative mass spectrometry‐based proteomic strategies in TNBC

Mass spectrometry‐based technologies offer a unique opportunity to profile cancer proteomes accurately and rapidly in terms of mass precision, sequencing speed, resolution, power and cost‐efficiency.^86^ Powerful mass spectrometers like Q‐TOF, TOF / TOF, Q‐OT and Q‐Exactive have high resolution, sensitivity and sub‐ppm mass accuracy, making them suitable for shotgun proteomics approaches to quantify hundreds to thousands of proteins in a biological sample.^87^, ^88^

The dynamic changes in cellular proteome abundance have a significant influence on different life processes. For example, the occurrence and development of many diseases are often accompanied by abnormal expression of specific proteins. Quantitative proteomics is the accurate quantification and identification of all proteins expressed in a genome or all proteins in a complex mixed system. The current quantitative proteomics technology is primarily divided into labelling (Label) and non‐labelling (Label Free) quantitative strategies, in which the labelling strategy is divided into in vivo labelling (such as SILAC^89^), and in vitro labelling (such as iTRAQ^90^ and TMT mark^91^).

### 3.3 Stable labelling approaches

The first global in‐depth proteomic analysis of TNBC molecular characteristics identified 12,000 distinct proteins whose expression patterns could discriminate between TNBC subtypes. This study also elucidated the specific TNBC pathway for metastasizing, adherence and angiogenesis.^92^ Different expression signatures for three proteins desmoplakin (DP), thrombospondin‐1 (TPS1) and tryptophanyl‐tRNA synthetase (TrpRS) were discovered for relapse and non‐relapse TBNC tumours using an iTRAQ labelling‐based proteomic approach.^93^ DP and TPS1 overexpression significantly altered disease‐free survival and increased the risk of TNBC patients’ recurrence. However, the overexpression of TrpRS was also reported to be associated with better disease‐free survival and lower recurrence risk. Another iTRAQ labelling‐based proteomics study identified several factors that have strong links with breast cancer molecular subtypes such as fibronectin, alpha‐2‐macroglobulin (A2 M), complement component‐4‐binding protein alpha (C4BPA) and complement factor‐B. One of these factors, the antiprotease A2 M, is an abundant plasma protein genetically modified and expressed differently in TNBC patients’ plasma and tissue samples.^94^

In 2010, Geiger et al. observed that the downregulation of PTEN in TNBC tumours resulted in higher activity of cell survival PI3K pathways. MCM5, STMN1, RCL1 and C9ORF114 were also found to be highly correlated with TNBC tumour cell growth.^95^

### 3.4 Label‐free proteomics strategies

Label‐free proteomics strategies in breast cancer research have been extensively used. Liu et al. examined 126 frozen TNBC primary tumours samples split into training and testing sets and identified a signature of 11 proteins linked with clinical outcome (CMPK1, AIFM1, FTH1, EML4, GANAB, CTNNA1, AP1G1, STX12, AP1M1, CAPZB and MTHFD1) by a label‐free proteomic approach. Of these 11 proteins, MTHFD1 was downregulated and associated with poor prognosis, while the other ten upregulated proteins led to a good prognosis in patients.^96^ Three of the upregulated proteins were involved in immunomodulation and apoptosis pathways, while MTHFD1 is involved in nucleotide and non‐coding RNA metabolism. Another proteomics study has further confirmed that FTH1, an immunomodulatory molecule involved in augmentation of CD8+ T cells in the tumour area, is a potential therapeutic target of TNBC.^97^ Sun et al. used label‐based proteomics to reveal that a tyrosine phosphatase called PTPN12 inhibited cellular transformation and metastasis of TNBC cells.^98^ He et al. also reported 30 proteins associated with drug resistance and poor patient survival of TNBC by using label‐free proteomics.^99^ Among these proteins, HSP70 kDa‐8, periostin, RhoA, actinin alpha 4, cathepsin D, preproprotein and annexin 1 were highly expressed in TNBC tumours resistant to neoadjuvant chemotherapy. The present study also identified three TNBC‐associated proteins (ALDH1A1, complementary component 1 inhibitor and G3BP). G3BP (also known as 90‐kDa Mac‐2‐binding protein), which was before unreported associated with TNBC, is a member of the beta‐galactoside‐binding protein family. Additionally, another study also identified ENO1 as a potential biomarker of TNBC.^100^

Phosphorylation is the most common type of post‐translation protein modification and some protein phosphorylation directly affects TNBC patients’ tumour progression. Semaan et al. identified several phosphoproteins associated with breast cancer progression by LC‐LTQ/FT‐ICR MS. Targeted dephosphorylation of such proteins, such as TRIM28, HSP90‐alpha, hnRNP A1, CLTC and myosin‐9, in breast cancer cells may inhibit TNBC progression. In the metastasis region, these phosphoproteins (TRIM28, HSP90‐alpha, hnRNP A1, CLTC and myosin‐9, HDGF) had no phosphopeptides and a higher number of phosphopeptides in the tumour site than in the normal tissue location. Increased phosphopeptides at the site of tumours suggest phosphorylation of these proteins that lead to a tumour phénotype are required for the onset of cancer in TNBC.^101^ Besides, HMGA1’s phosphorylation activated the IL4‐mediated signalling pathway and enhanced the TNBC tumour's metastatic potential.^101^ Using a quantitative phosphoproteomic approach based on SILAC, Wu et al. assessed tyrosine kinase activity in TNBC and have discovered that the receptor tyrosine kinase (AXL) is activated in most invasive TNBC cells, and the positive expression of AXL significantly reduces the patients’ survival.^102^ Their phosphoproteomic analysis also showed that AXL was highly phosphorylated in TNBC. Upon activation (phosphorylation) AXL dimerized with the receptor MET, resulting in activation of downstream signalling pathways (PI3K‐AKT and FAK‐SRC), augmenting cell proliferation and migration in TNBC.

Due to its high sensitivity, precision, reproducibility and performance in biomedical research, proteomics methods have become practical investigative tools. MS‐based proteomic approaches, both labelled‐free and isobaric, allow thousands of proteins to be profiled globally across biological conditions and used to uncover TNBC's potential protein biomarkers.

## 4 CONCLUSION AND PERSPECTIVE

The development of high‐profile sequencing technologies and computational analysis tools, including transcriptomic and proteomic technologies, has enhanced our understanding of TNBC. Transcriptomic analyses have provided a considerable amount of information on the gene expression patterns in breast cancer. For clinical applications, transcriptomic can be employed to classify TNBC into unique molecular subtypes and to propose reliable therapeutic targets, and large‐scale approaches such as proteomics can be used to decipher the global picture of TNBC cancer biology.

At present, the transcriptomic and proteomic prognostic studies on TNBC can be basically divided into two categories: one is to establish a scoring model to identify prognosis; the other is to study the prognostic relevance through a single indicator involved in signalling pathways. In the transcriptome study, considering the interaction among mRNA, lncRNA, miRNA and circRNA from multiple perspectives, researchers attempted to establish a ceRNA regulatory network to evaluate the prognostic characteristics of tumours.^20^, ^33^, ^103^ However, due to big data modelling, analysis tool selection, tumour heterogeneity and other reasons, the final feature scoring models are different. Adopting uniform standards and developing specific analytical data sets and tools can help balance cluttered data to a certain extent, particularly for cross‐omics research. In proteomic studies, the integration and analysis of gene data with protein‐related information based on immunohistochemistry, lc‐ms /MS and other technologies are one of the most important means of proteomics research, which helps discover potential targets, signalling pathways within or between tumours, and overall tissue biological characteristics.

Multi‐omic data's emergence has become a routine in cancer studies. However, the challenge is increasingly difficult to assimilate the rapidly growing number of ‘big data’. Intelligent utilization and management of these data require massive computational resources and accurate statistical methodologies to unearth the hidden links among different sub‐components.

The multiple layers of cancer biology are detailed in multi‐omic data, but our perception of the nature of cancer seems confusing with the endless complexity. Enormous efforts must be made to acquire a considerable quantity of multi‐omic data indicating the diverse biological signatures of the development of TNBC.

The treatment of TNBC remains to be challenging since its poor patient outcomes and few therapeutic targets. A better understanding of TNBC carcinogenesis is a prerequisite for more sophisticated TNBC subtyping and the development of personalized treatment options.

This paper reviews the recent advances in TNBC research through transcriptomics and proteomics and understands that the power of proteomics and transcriptomics can be used for decoding the complexity of TNBC, which will develop more effective clinical interactions. It is high time we take advantage of these abundant resources to unveil TNBC, and we hope that this intractable cancer will be precisely targeted soon.

## CONFLICT OF INTERESTS

The authors declare that they have no competing interests.

## AUTHOR CONTRIBUTION

**Yuan Li:** Writing – original draft (equal). **Xiangyi Kong:** Writing – review & editing (equal). **Lixue Xuan:** Conceptualization (equal); Supervision (equal). **Zhongzhao Wang:** Investigation (equal).

## Supporting information

Figure S1

Figure S2

Figure S3

Table S1

Table S2
