# Supplementary figures and images for: Recent advances of transcriptomics and proteomics in triple‐negative breast cancer prognosis assessment
Source: J Cell Mol Med. 2022 Feb 11;26(5):1351–62. doi: 10.1111/jcmm.17124 (PMC8899180; doi:10.1111/jcmm.17124)

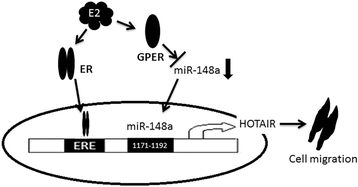

Supplement: Supplementary file 1 — Figure S1 [file JCMM-26-1351-s005.gif]

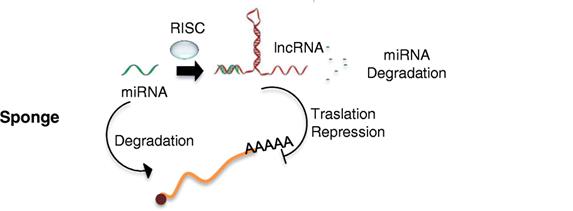

Supplement: Supplementary file 2 — Figure S2 [file JCMM-26-1351-s002.jpg]

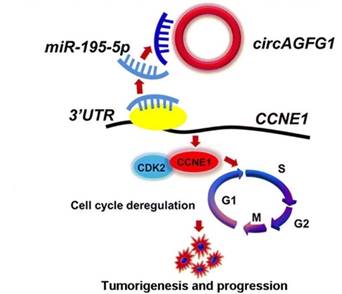

Supplement: Supplementary file 3 — Figure S3 [file JCMM-26-1351-s004.jpg]
